# Neurovascular crossing patterns between leash of Henry and deep branch of radial nerve: implications for neurointervention and diagnostic imaging

**DOI:** 10.1007/s00256-024-04740-1

**Published:** 2024-07-31

**Authors:** Aurea V. R. Mohana-Borges, Livia Tavane S. D. Silva, Ronaldo S. Mohana-Borges, Sheronda Statum, Saeed Jerban, Yuanshan Wu, Victor Barrère, Sergio A. L. Souza, Christine B. Chung

**Affiliations:** 1 Radiology, Federal University of Rio de Janeiro, Rio de Janeiro, Brazil; 2 Radiology, University of California, San Diego, USA; 3 Veterans Affairs Medical Center, San Diego, USA; 4 Laboratory of Biotechnology and Structural Bioengineering, Federal University of Rio de Janeiro, Rio de Janeiro, Brazil; 5 Department of Orthopaedic Surgery, University of California, San Diego, CA USA; 6 Department of Bioengineering, University of California, San Diego, CA USA; 7 Research Service, VA San Diego Healthcare System, San Diego, CA USA

**Keywords:** Ultrasound, High-resolution ultrasound, Magnetic resonance imaging, Leash of Henry, Deep branch of the radial nerve, Posterior interosseous nerve, Hydrodissection

## Abstract

**Objective:**

To detail the neurovascular crossing patterns between the leash of Henry (LoH) and the deep branch of the radial nerve (DBRN) in supination and pronation of the forearm, using imaging methods with anatomic correlation.

**Materials and methods:**

This cross-sectional study was performed ex vivo with HRUS and MRI with anatomic correlation on 6 samples and in vivo with HRUS with Doppler on 55 participants scanned bilaterally. The in vivo participants were enrolled over a 6-month period. The crossing patterns between the LoH and DBRN were assessed ex vivo and in vivo. Additional morphological features of the DBRN, LoH, and fat plane were assessed in vivo only. Biometric features of the participants were recorded. Statistical analyses were performed using Shapiro–Wilk, parametric and non-parametric tests.

**Results:**

The most common neurovascular crossing pattern was the ascending branch of the radial recurrent artery (RRAab) crossing below (ex vivo: 83.3%, in vivo: 85.3%) and the muscular branch crossing above (ex vivo: 100%, in vivo: 63.2% %) the DBRN. Both the deep and superficial surfaces of the DBRN exhibited an intimate relationship with the vessels of the LoH. A positive correlation between vessel diameter and anthropometric factors was observed. In addition, the muscular branch exhibited a significantly smaller diameter than the RRAab.

**Conclusion:**

Our study detailed the relationship between the LoH and the DBRN and highlighted the high incidence of vessel crossing above the DBRN at the level of the muscular branch. Knowledge of neurovascular crossings is crucial for understanding neurovascular entrapment syndromes and planning interventional procedures to reduce vascular complications.

**Supplementary Information:**

The online version contains supplementary material available at 10.1007/s00256-024-04740-1.

## Introduction

The leash of Henry (LoH) is a terminology coined in the past century after Dr. Arnold Henry drew attention to the surgical importance of the branches of the radial recurrent artery (RRA) for surgical access to the elbow and proximal forearm [1]. Despite Dr. Henry’s pioneering description, we still lack detailed information about the relationship between the LoH and nearby neural structures. This knowledge gap is further accentuated when considering the dynamic nature of the nerve-vessel relationship, particularly in assessing neurovascular crossings under various forearm positions. The LoH has been implicated in nerve compression syndromes, including the radial tunnel and posterior interosseous nerve (PIN) syndromes, with emphasis placed on the relationship between the LoH and the deep branch of the radial nerve (DBRN).

One proposed mechanism for neurovascular compressive syndromes involves mechanical nerve irritation due to direct contact with the blood vessels. This nerve irritation leads to a focal area of demyelination, neuroinflammation, and the appearance of Renault bodies, with structural changes often predominating at the nerve’s edge and subperineurial space [2–6]. Hence, mapping the LoH neurovascular crossings can contribute to identifying areas prone to structural changes, neuroinflammation, and partial fascicular lesions, thereby improving diagnostic accuracy.

High-resolution ultrasound (HRUS) and magnetic resonance imaging (MRI) have been increasingly used to assess peripheral nerves, one complementing the other in the diagnoses of nerve pathology [7–9]. HRUS has demonstrated several advantages, including excellent spatial resolution of superficial nerves, dynamic real-time imaging capabilities, non-invasive nature, and excellent cost-effectiveness. Moreover, the combination of HRUS with Doppler technology has been used to discriminate small nerves from small vessels, enabling a detailed assessment of the neurovascular structures [7]. Conversely, MRI has excellent soft tissue contrast, providing additional information about the surrounding soft tissues, such as muscle and perineural fat. However, some disadvantages of the techniques include dependence on operator experience in performing nerve HRUS and the high cost of MRI.

The purpose of our study was to detail the neurovascular crossing patterns between the LoH and the DBRN in supination and pronation of the forearm, using HRUS, color Doppler, and MRI with anatomic correlation. Based on clinical practice, we hypothesize a predominant vascular pattern where the ascending branch of the radial recurrent artery (RRAab) crosses below and the transverse muscular branch crosses above the DBRN.

## Material and methods

### Ex vivo study with HRUS, MRI, and anatomical correlation

#### Specimens

Six upper limbs were obtained from five fresh human cadavers from the University of California San Diego anatomic laboratory. They were frozen at − 80 °C (Forma Bio-Freezer; FormaScientific, Marietta, OH) and thawed at room temperature for 24–48 h before HRUS and MRI.

#### HRUS and MRI

HRUS of the elbow and proximal forearm focused on the radial tunnel was conducted on five out of six specimens by a single musculoskeletal radiologist (A.M.B.) with more than 20 years of experience. The examinations were performed using a VEVO MD machine with transducers with 22 MHz and 48 MHz nominal frequencies. The focus was adjusted to the level of the DBRN, and the depth was tailored to the individual characteristics of each specimen. The specimens were scanned with the forearm in supination and then in pronation. The radiologist saved anonymized cine clips onto a pen drive for subsequent offline analysis.

High-resolution MRI of the elbow and proximal forearm focused on the radial tunnel was performed on six specimens using a 3.0-T scanner (Discovery MR750, GE Healthcare Technologies, Milwaukee, WI, USA). The specimens were placed inside the coil with the forearm in supination and then in pronation. The MRI protocol included images in the axial plane obtained with the following sequences: T1-weighted (TR = 600–850 ms, TE = 15 ms, slice thickness = 2 mm, spacing = 2.6 mm, matrix = 384 × 384), T2-weighted with fat saturation (fat sat) (TR = 3.000–7.500 ms, TE = 64–72 ms, slice thickness = 2 mm, spacing = 2.6 mm, matrix = 384 × 384 or 420 × 420), and 3D isotropic T1 CUBE (TR = 602–902 ms, TE = 18–29 ms, slice thickness = 0.6 × 0.6 mm, spacing = 0.6 mm, matrix = 256 × 256 or 400 × 400).

#### Preparation of anatomic sections and sample dissection

After imaging, samples were frozen at − 80 °C with the forearm either in supination (*N* = 3) or pronation (*N* = 3). Subsequently, five samples were sectioned in the axial plane with a diamond blade (EXAKT 312 Pathology Saw, EXAKT Technologies, Inc, Oklahoma City, OK), resulting in slices with a 3-mm thickness. These slices were rinsed with running water and photographed for macroscopic inspection. One specimen was thawed at room temperature for 24–48 h and dissected via an anterior approach.

### In vivo study with HRUS and Doppler

#### Study design and participants

This cross-sectional study received approval from the Federal University of Rio de Janeiro Institutional Review Board. Written informed consent was obtained from all individual participants included in the study. HRUS of the DBRN with Doppler was performed bilaterally on 55 asymptomatic volunteers, with the forearm positioned in both supination and pronation, resulting in a total of 110 nerves eligible for the study.

Inclusion criteria were asymptomatic volunteers of any sex over 15 years of age. Exclusion criteria were (a) symptoms that compromise sustained forearm supination and pronation, (b) functional deficit in the forearm range of motion (ROM), (c) previous history of interventional procedure or surgery in the radial tunnel and elbow, (d) incidental masses compressing the nerve, (e) exams with the Doppler unavailable for analysis, and (f) undetectability of the DBRN and/or vessels of the LoH.

A bioimpedance foot scale (Relaxmedic®) was used for measurements of weight, body mass index (BMI), percentage of muscle mass, and percentage of total body fat. BMI was divided into categories as follows: underweight < 18.5, normal weight = 18.5–24.9, overweight = 25–29.9, obesity class I = 30–34.9, obesity class II = 35–39.9, and obesity class III ≥ 40. Hand dominance and height were recorded based on subjects’ reports.

#### Imaging protocol: HRUS and color Doppler

HRUS of the elbow and proximal forearm focused on the radial tunnel was performed with an 18–5 MHz linear transducer (Philips, Affiniti 50) by a single musculoskeletal radiologist (A.M.B.). The focus was adjusted to the level of the DBRN, and the depth was tailored to the individual characteristics of each participant. During the exam, participants sat in a comfortable chair with their forearms extended and supported on a table, which was adjusted to place the shoulder in a 90° flexed position. A straight splint surrounded by fixation straps was used on the participants’ hands and wrists for partial immobilization. The radiologist saved the B-mode and Doppler cine clips on a pen drive for offline anonymization and analysis.

#### Image analysis *(*ex vivo and in vivo*)*

Two radiologists (A.M.B. and L.T.S., the latter with more than 10 years of experience) analyzed the ex vivo and in vivo images in consensus to determine the crossing patterns between the RRAab, RRA muscular branch, and DBRN. The analysis was performed from the origin of the DBRN to the superior arcade of the supinator muscle (SASM). Vascular crossings occurring within the supinator tunnel were not included in the analysis.

**Fig. 1:**
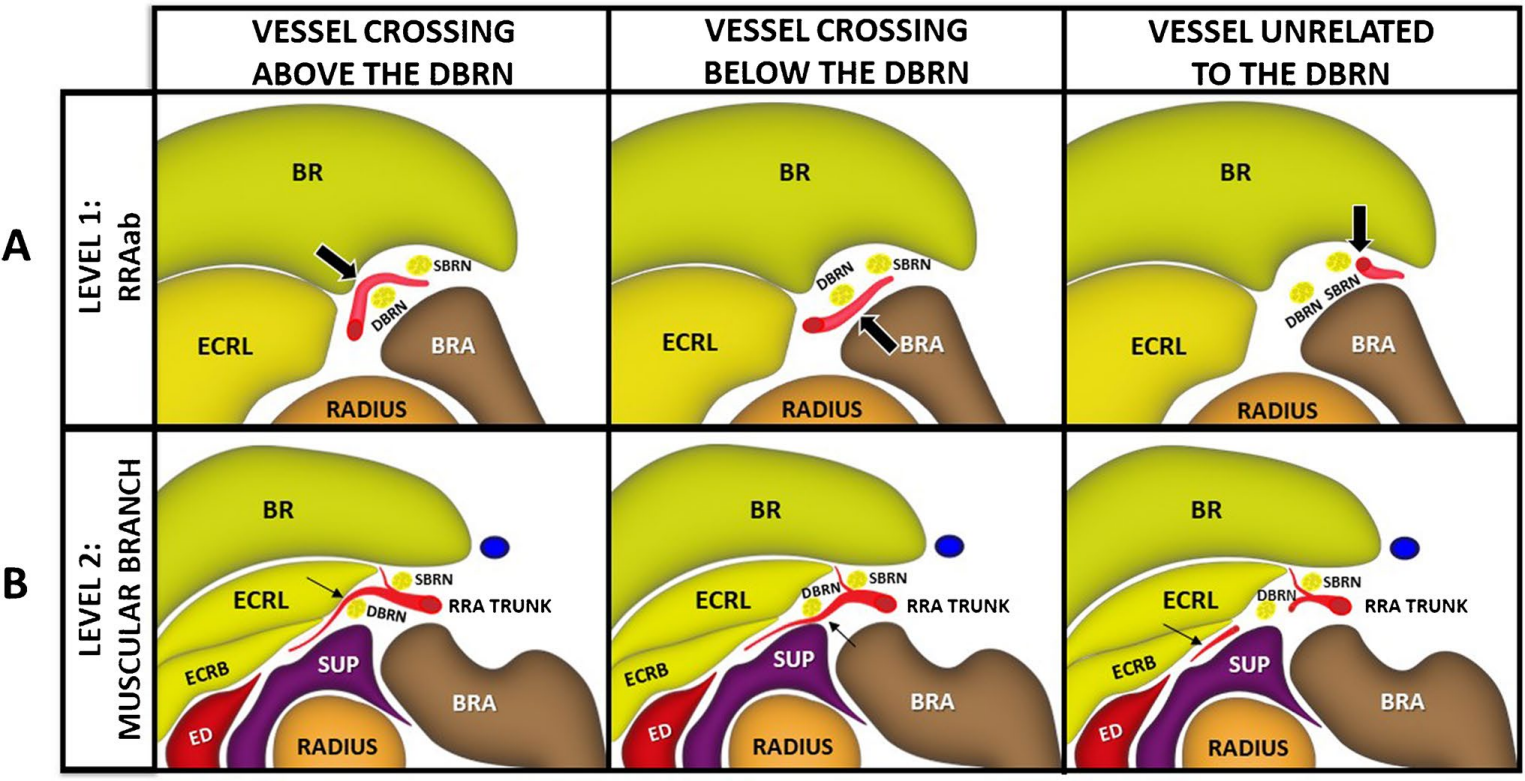
Vascular crossing patterns between the leash of Henry and the deep branch of the radial nerve (DBRN). **A** Level of the ascending branch of the radial recurrent artery (RRAab) (large arrow). **B** Level of the muscular branch (thin arrow). BR, brachioradialis muscle; BRA, brachialis muscle; ECRB, extensor carpi radialis brevis muscle; ECRL, extensor carpi radialis longus muscle; ED, extensor digitorum muscle; RRA TRUNK, radial recurrent artery trunk; SBRN, superficial branch of the radial nerve; SUP, supinator muscle

**Fig. 2:**
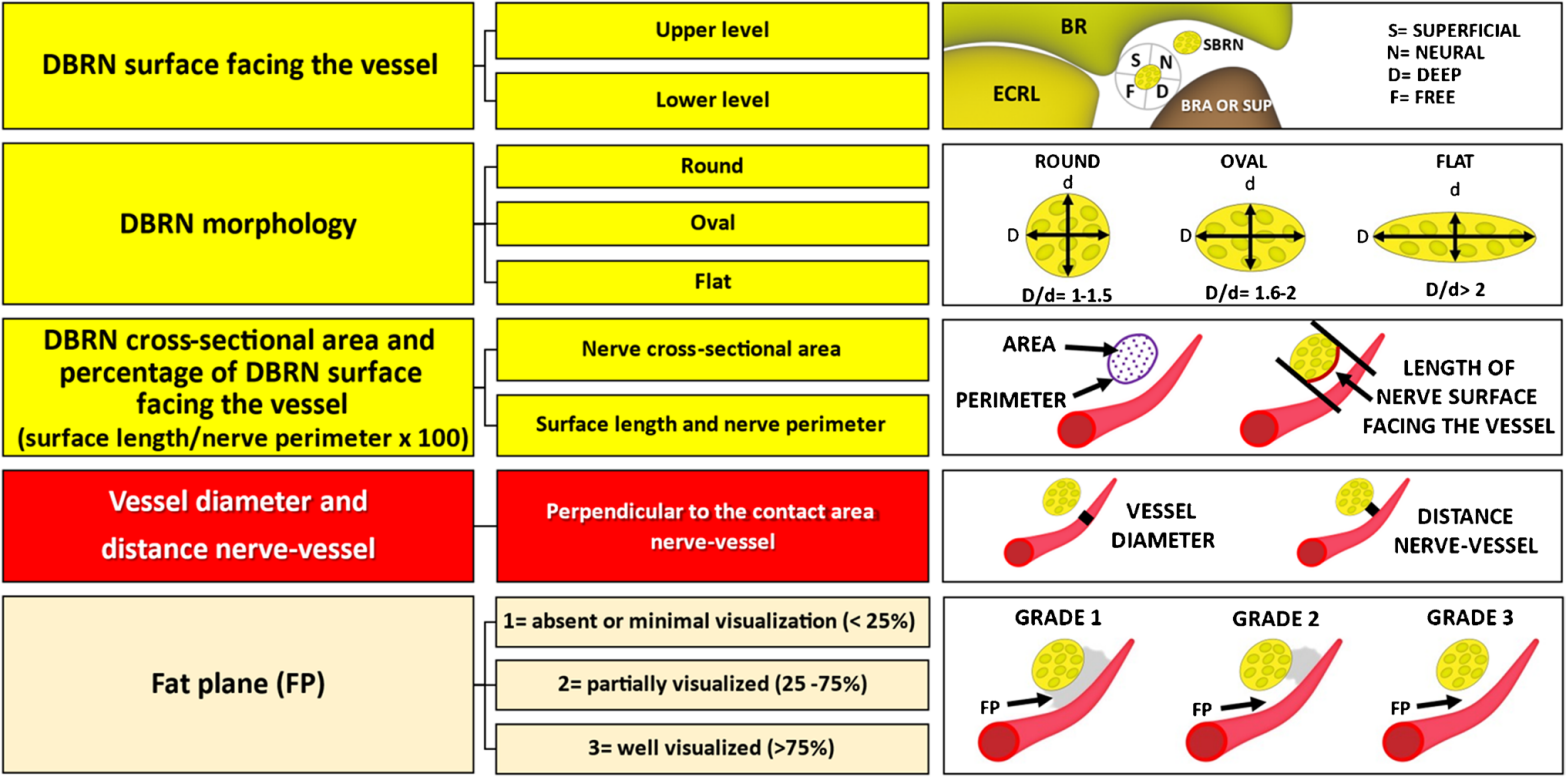
B-mode analysis of the relationship between LoH and DBRN. BR, brachioradialis; BRA, brachialis; ECRL, extensor carpi radialis longus; SUP, supinator

The vascular crossings were classified as either above or below the DBRN. In cases where no vessel was observed crossing the nerve, it was classified as unrelated (Fig. 1). The reference for the classification was the forearm’s anterior and lateral surfaces in supination and pronation. Subsequently, the following parameters were assessed in B-mode for the in vivo images only: (1) surface of the DBRN facing the vessel, (2) DBRN morphology (round/oval/flat), (3) DBRN cross-sectional area (mm), (4) percentage of the DBRN surface in contact with the vessel (nerve length in contact with the vessel divided by the nerve perimeter multiplied by 100), (5) vessel diameter (mm), (6) distance nerve-vessel (mm), and (7) fat plane between the vessel and DBRN (1 = minimal or absent, 2 = partially defined, 3 = well-defined) (Fig. 2). These parameters were not assessed in ex vivo images due to potential biases introduced by freezing and thawing cycles, as well as the lack of blood flow and intrinsic distension of the vessels in the specimens.

The nerve surface was classified as superficial, deep, neural, and free based on the anatomical landmarks of soft tissues accessible by HRUS, such adjacent muscles and the superficial branch of the radial nerve (SBRN) (Fig. 2). Specifically, the superficial surface of the DBRN faced the muscles of the lateral compartment (brachioradialis and extensor carpi radialis longus and brevis), while the deep surface faced deep muscles in the elbow and proximal forearm (brachialis and supinator). The superficial and deep surfaces were oriented slightly laterally and medially, respectively. Finally, the neural surface faced the SBRN, and the free surface was opposite the neural surface.

The classification of the nerve morphology was based on the ratio between the widest and narrowest nerve diameters [10]. The nerve was classified as round when the ratio was 1.0 to 1.5, oval when the ratio was between 1.6 and 2.0, and flat when the ratio was greater than 2.0 (Fig. 2). The imaging analysis was performed after a training session using cine clips of 20 participants. All disagreements were resolved by consensus.

#### Statistical analysis

Statistical analysis and graphs were performed using the open-source Jamovi software (2.2.2 version) and Excel 365. The normality of the data was assessed by using the Shapiro–Wilk test. Parametric data were presented as the mean ± standard deviation, and non-parametric data as the median and interquartile range (IQR). Welch’s test was used to estimate unequal variance of age and sex. A paired *t*-test was employed to compare the variables in supination and pronation and Kruskal–Wallis test to compare B-mode variables across different vessels of the LoH and in various forearm positions. Spearman’s rho was applied to measure the correlation of multiple variables. Two-tailed *p* < 0.05 was considered to indicate a statistically significant difference.

## Results

### Ex vivo study with HRUS, MRI, and anatomical correlation

#### Specimen characteristics

Donor data is listed in Table 1. There were 4 males (right side) and 1 female (both sides), age at death: 41–54 years, mean: 49.4 years. Five specimens showed a normal DBRN appearance on imaging and anatomical sections. One specimen exhibited signs of DBRN neuropathy at the level of the arcade of Frohse. Concurrently, this specimen displayed almost complete obliteration of the perineural fatty plane on the T1-weighted sequences, apparently due to a combination of muscle hypertrophy and tissue edema.

**Table 1:** Specimen characteristics

Specimen	Sex	Age (y)	Weight (kg)	Height (m)	BMI	Side
1	Male	54	73.5	1.70	25	Right
2	Male	41	72.6	1.85	21	Right
3	Female	53	108.9	1.88	31	Left
4	Male	51	63.5	1.80	20	Right
5	Female	53	108.9	1.88	31	Right
6	Male	48	90.7	1.73	30	Right

#### Image analysis

**Fig. 3:**
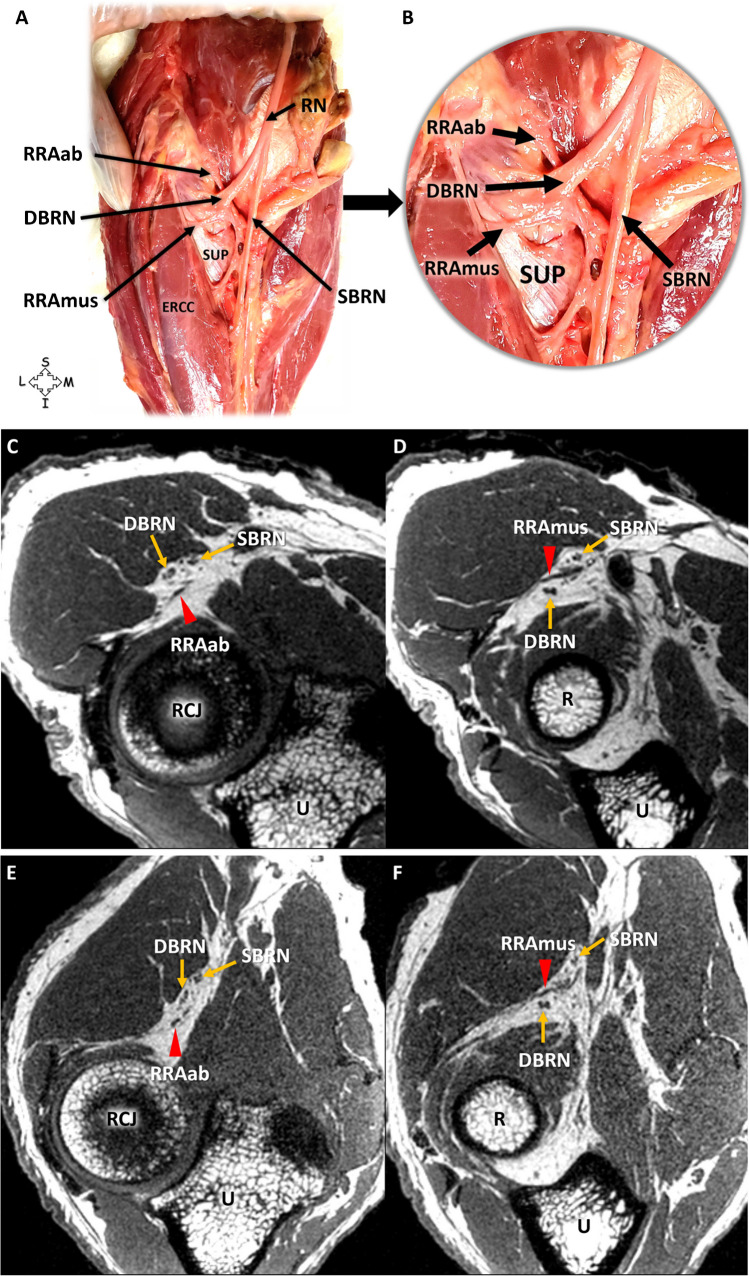
Relationship between the branches of the radial nerve (RN) and vessels of the leash of Henry in a 48-year-old male cadaveric specimen. **A**, **B** Anatomic dissection. **B** Magnified view. **C**, **D**, **E**, **F** MRI of the elbow and proximal forearm in supination with T1-weighted 3D CUBE sequence in the axial plane. **C**, **D** Supination. **E**, **F** Pronation. Note the ascending branch of the radial recurrent artery (RRAab) crossing below (**A**, **B**, **C**) and the muscular branch (RRAmus) crossing above (**A**, **B**, **D)** the deep branch of the radial nerve (DBRN). **C**, **D**, **E**, **F** In the T1-weighted Cube sequence, the fat pad of the radial tunnel presents high signal intensity, with local fat serving as a protective layer interposed between the vessel and the nerve. ECRB, extensor carpi radialis brevis muscle; SBRN, superficial branch of the radial nerve; SUP, supinator muscle; R, radius; RCJ, radiocapitellar joint; U, ulna

**Fig. 4:**
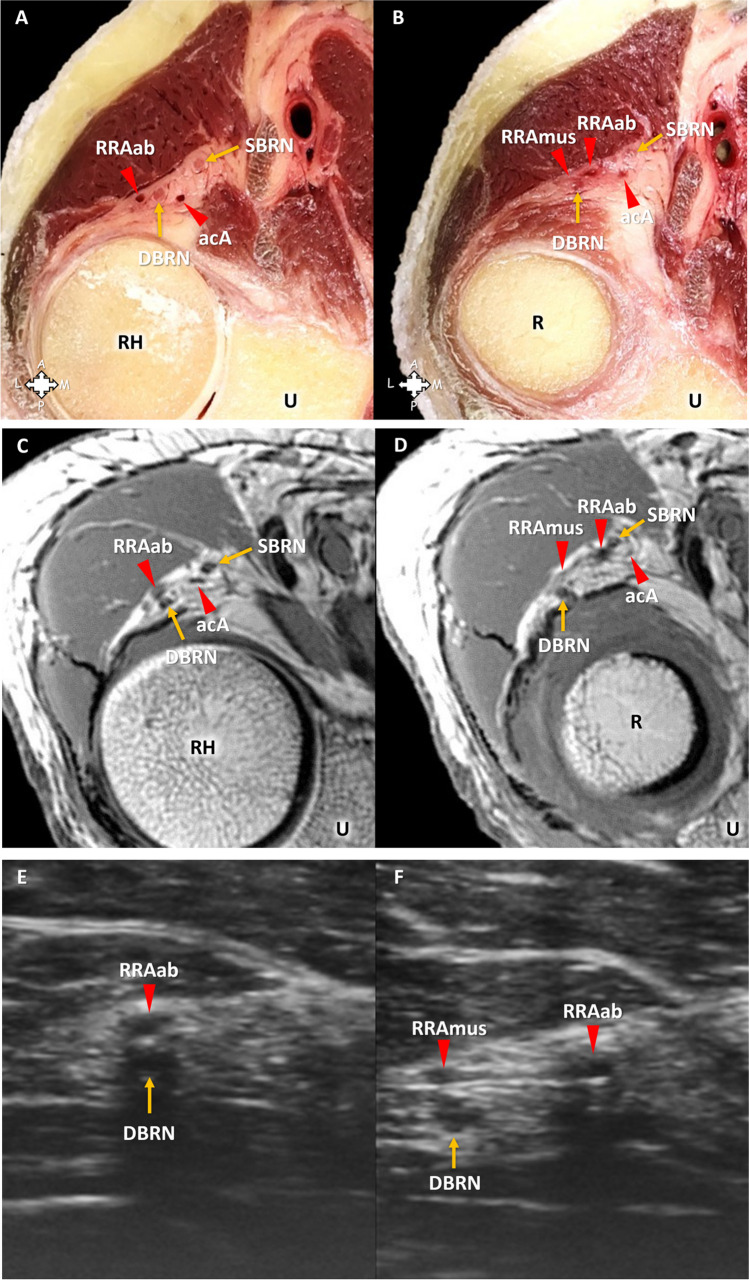
Anatomic variation of the vessels of the leash of Henry in a 41-year-old male cadaveric specimen. **A**, **B** Anatomic section. **C**, **D** MRI with T1-weighted sequence. **E**, **F** HRUS with 48 MHz transducer. **A**, **B**, **C**, **D**, **E**, **F** Elbow and proximal forearm in supination. **A**, **B**, **C**, **D** Axial plane. **E**, **F** Short axis of the deep branch of the radial nerve (DBRN). **A**, **C**, **E** The ascending branch of the radial recurrent artery (RRAab) crosses above the DBRN. **A**, **C** Note an accessory artery (acA) near the branches of the radial nerve. This accessory artery crosses below the DBRN and anastomoses with the RRAab near the radial recurrent artery trunk (not shown). **B**, **D**, **F** In this specimen with anatomic variation, the muscular branch of the radial recurrent artery (RRAmus) branches off the RRAab. SBRN, superficial branch of the radial nerve; R, radius; RH, radial head; U, ulna

The most common neurovascular crossing pattern was the RRAab crossing below and the muscular branch crossing above the DBRN (Fig. 3). The crossing between the RRAab and the DBRN was observed to occur below the nerve in five specimens (83.3%), while in one specimen, it was noted to occur above (16.7%). Notably, in the specimen where the RRAab crossed above the DBRN, an accessory artery was observed crossing below the nerve, forming an anastomosis with the RRAab near the trunk of the RRA (Fig. 4). Across all specimens, one muscular branch was consistently seen crossing above the DBRN (100%) and coursing through the intermuscular plane between the extensor carpi radialis and supinator muscles. The muscular branch of the RRA was found between the branches of the radial nerve, with the DBRN located inferiorly and the SBRN located superiorly. However, in one specimen with signs of DBRN neuropathy and soft tissue edema, the visualization of the muscular branch was compromised on MRI. Nonetheless, the muscular branch was well visualized on HRUS with the 48 MHz transducer.

### In vivo study with HRUS and Doppler

#### Participant characteristics

**Fig. 5:**
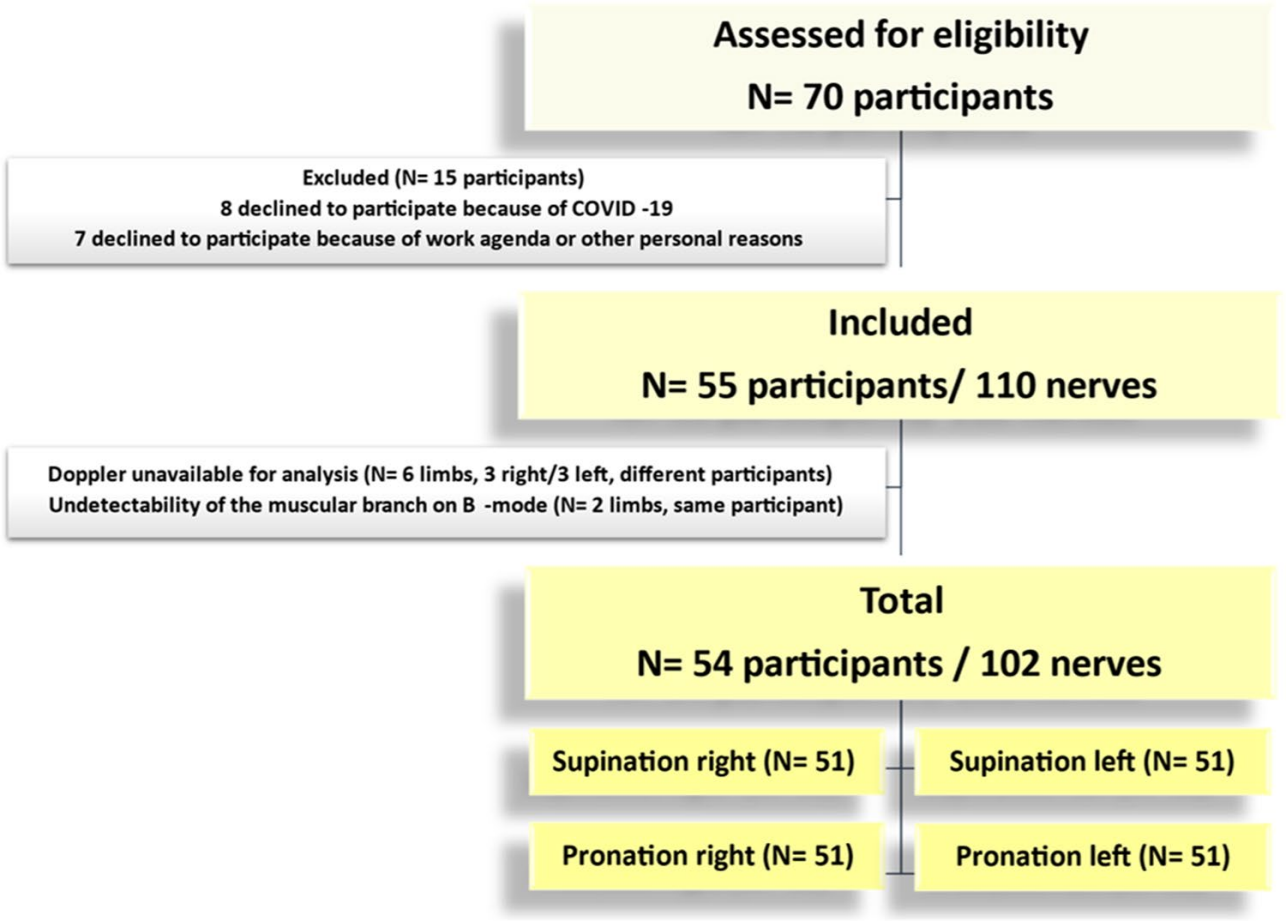
Flowchart shows participant selection process

The participant selection flowchart is shown in Fig. 5, and the participant data is presented in Table 2. The study population consisted of 102 nerves from 54 asymptomatic participants (median age, 36.0 years; interquartile range [IQR], 23.2–51.0 years; age range, 16–63 years; 28 [51.9%] women), with 48 of them having bilateral assessment. Eight neurovascular crossings were excluded from analysis due to the unavailability of Doppler imaging (*N* = 6 limbs, 3 right sides/3 left sides) and the inability to detect the muscular branch in B-mode (*N* = 2 limbs from one participant). Age was not significantly different between women (median 35.5 years, IQR, 23.8–50.3 years) and men (median 36.5 years, IQR, 22.8–52.8 years) with *p* = 0.74. Among participants with BMI calculated (*N* = 53), one (1.9%) was underweight, 19 (35.8%) were normal in weight, 16 (30.2%) were overweight, and 17 (32.1%) were obese. In the obese group (*N* = 17), the majority had obesity class I (*N* = 14, 82.3%).

**Table 2:** Participant characteristics

Parameter	*N* (%)	Mean (± SD)	Median (IQR)	Shapiro–Wilk *w*/*p*
Age (y)	54 (100)	…	36 (23.2–51.0)	0.921/0.002
Sex	54 (100)	…	…	…
Female	28 (51.9)	…	…	…
Male	26 (48.1)	…	…	…
Weight (kg)*	53 (98.1)	78.2 (± 19.17)	…	0.957/0.053
Height (m)	54 (100)	1.69 (± 0.08)	…	0.982/0.595
BMI (kg/m^2^)*	53 (98.1)	27.1 (± 5.44)	…	0.964/0.108
Muscle mass**	52 (96.3)	35.4 (± 4.79)	…	0.974/0.303
Total body fat**	52 (96.3)	29.8 (± 8.62)	…	0.981/0.576
Hand dominance	54 (100)	…	…	…
Right	51 (94.4)	…	…	…
Left	3 (5.6)	…	…	…

*One participant refused to measure his weight. **In one additional participant, the scale calculated subcutaneous fat but could not calculate muscle mass and total body fat

#### Image analysis

**Fig. 6:**
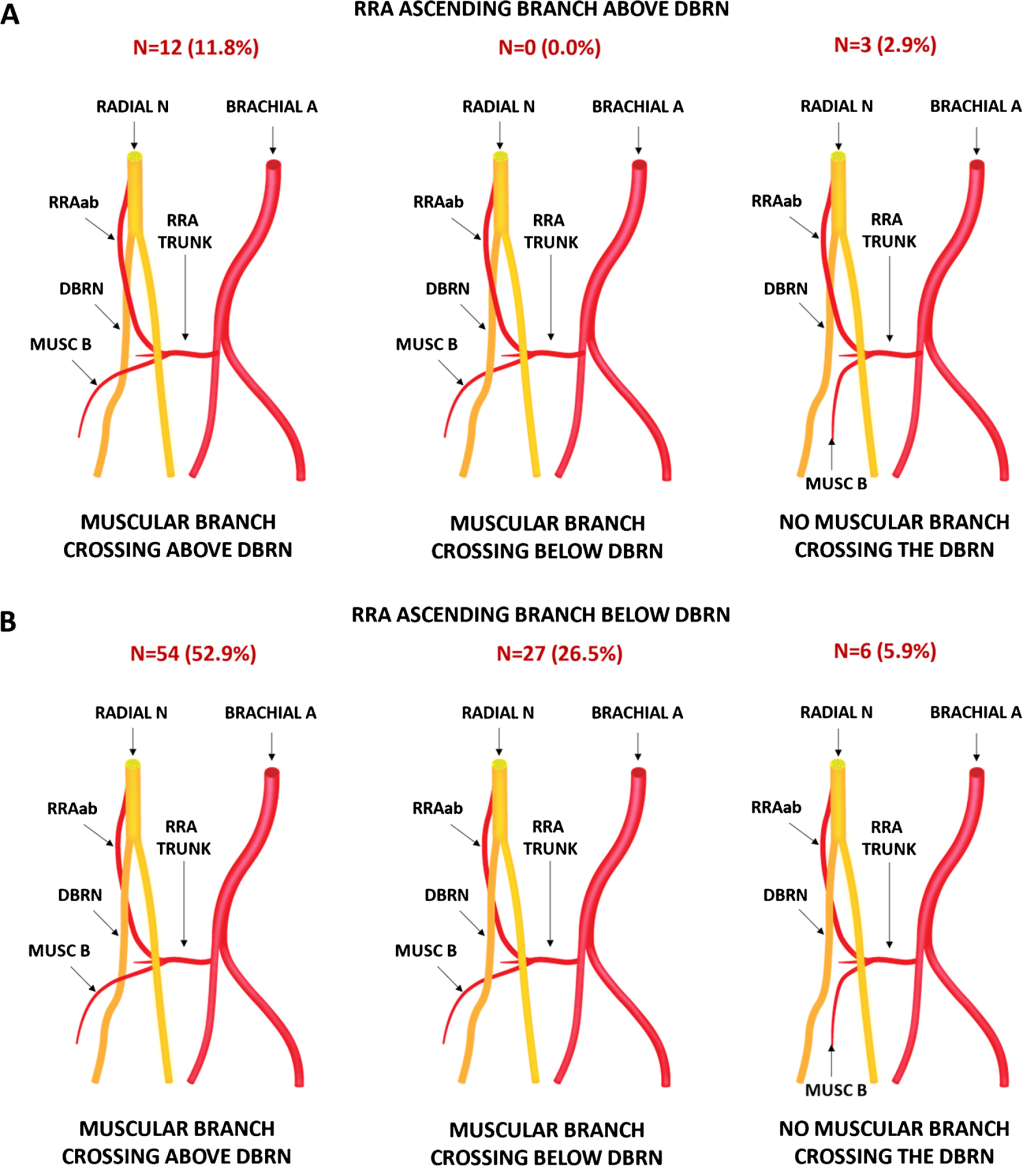

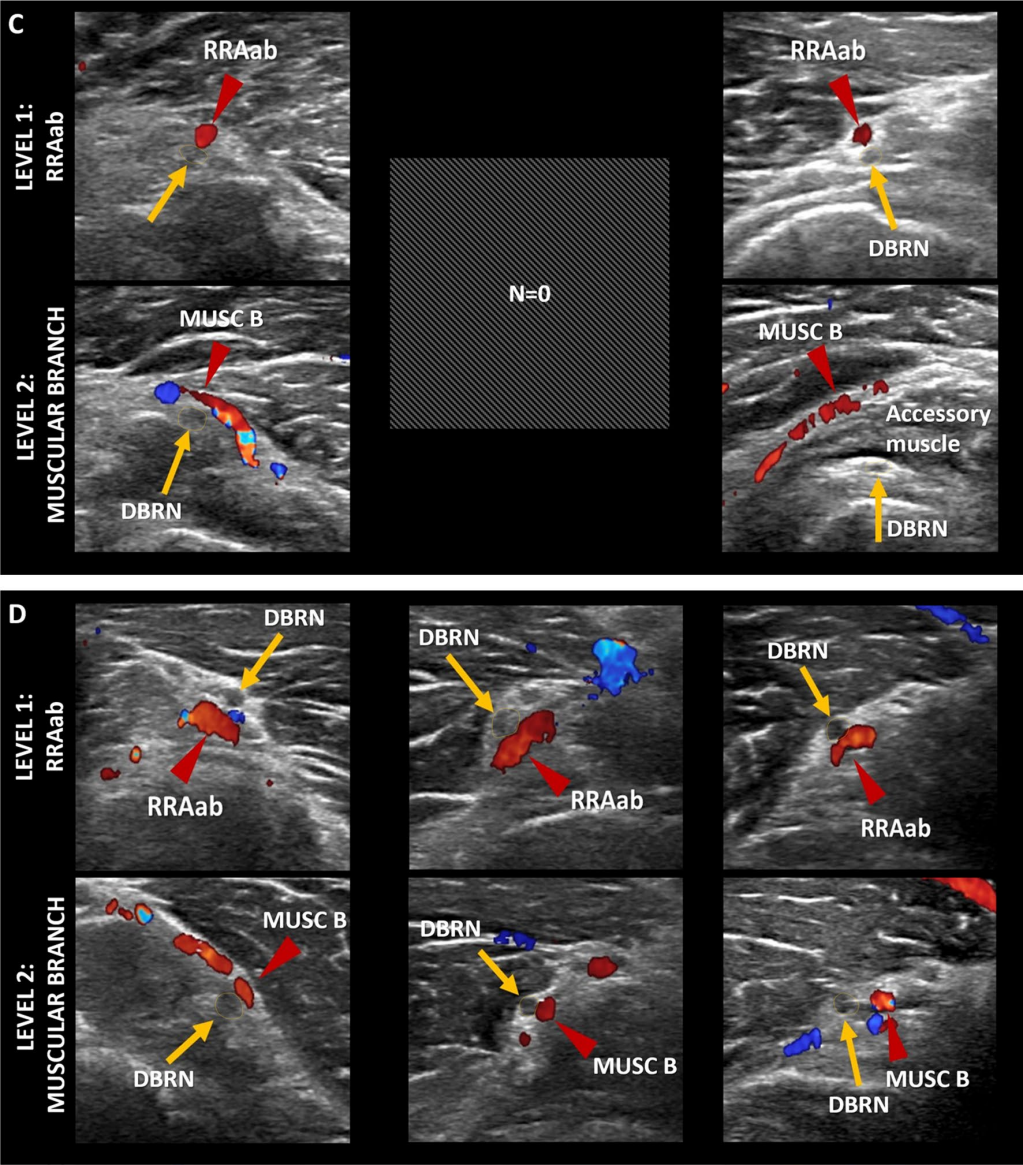
Crossing patterns between the leash of Henry and the deep branch of the radial nerve (DBRN). **A**, **B** Diagrams. **C**, **D** Corresponding HRUS with color Doppler. MUSC B, muscular branch; RRAab, ascending branch of the radial recurrent artery

**Fig. 7:**
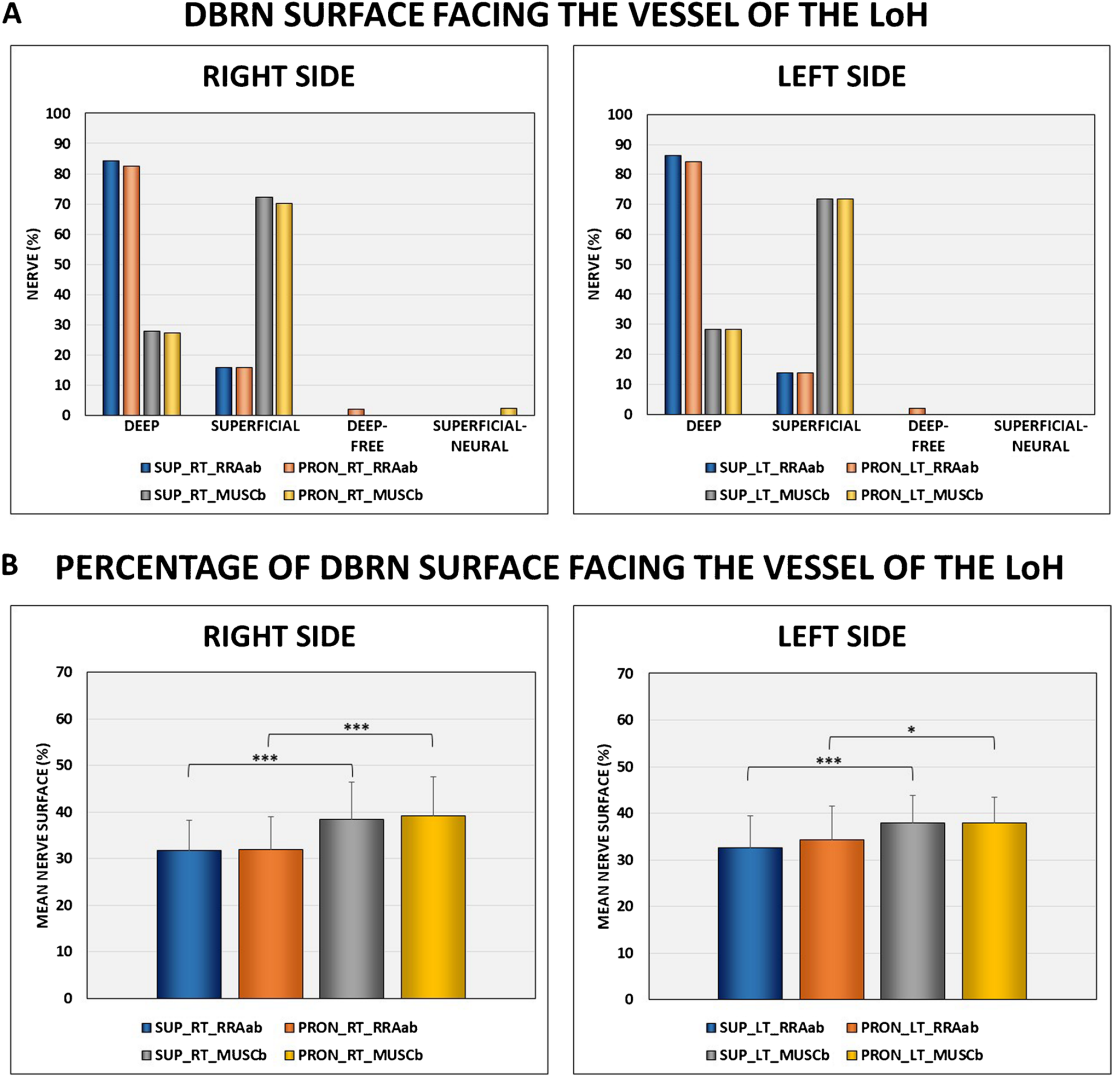

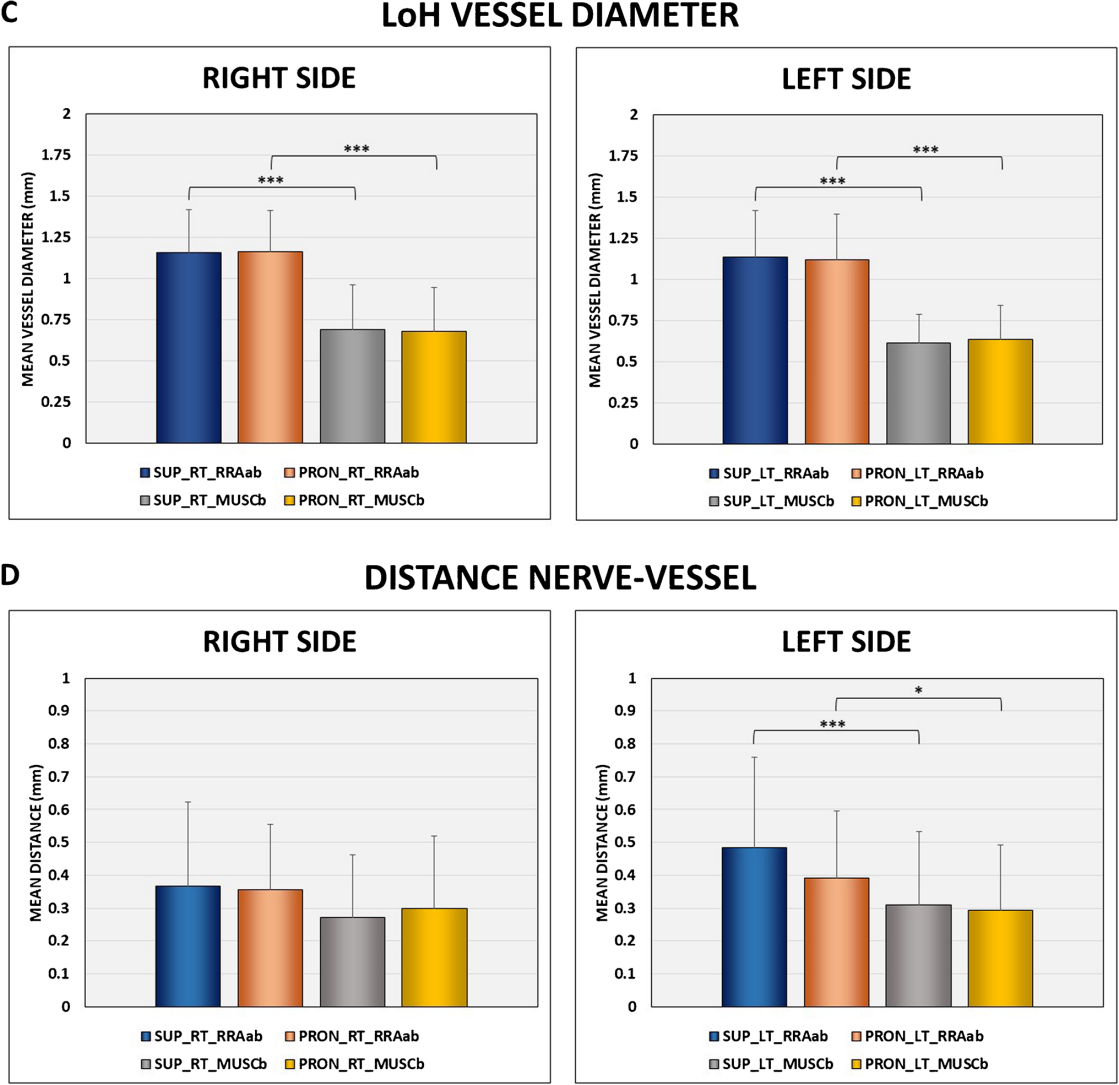
B-mode analysis of the relationship between LoH and DBRN on the right and left sides. **A** DBRN surface facing the vessel of the LoH.** B** Percentage of DBRN surface facing the vessel of the LoH.** C** LoH vessel diameter. **D** Distance nerve-vessel. SUP_RT_RRAab, supination right at the level of the ascending branch of the radial recurrent artery; PRON_RT_RRAab, pronation right at the level of the ascending branch of the radial recurrent artery; SUP_RT_MUSCb, supination right at the level of the muscular branch; PRON_RT_MUSCb, pronation right at the level of the muscular branch; SUP_LT_RRAab, supination left at the level of the ascending branch of the radial recurrent artery; PRON_LT_RRAab, pronation left at the level of the ascending branch of the radial recurrent artery; SUP_LT_MUSCb, supination left at the level of the muscular branch; PRON_LT_MUSCb, pronation left at the level of the muscular branch

The neurovascular crossings were as follows: (a) RRAab above DBRN = 15 (14.7%) and muscular branch above = 12 (11.8%), below = 0 (0%), and unrelated = 3 (2.9%), and (b) RRAab below DBRN = 87 (85.3%) and muscular branch above = 54 (52.9%), below = 27 (26.5%), and unrelated = 6 (5.9%) (Fig. 6). Most nerves have the deep surface facing the RRAab (approximately 84% on the right side and 86% on the left side) and the superficial surface facing the muscular branch (approximately 72% on both sides) (Fig. 7A). There was a tendency to change the morphology of the nerve from round to flatter by changing the forearm position from supination to pronation, as well as by changing the level of the vessel of the LoH under analysis (specifically from the RRAab to the muscular branch). No significant changes were noted in the cross-sectional area of the DBRN by changing the forearm position from supination to pronation at the level of the RRAab and the muscular branches bilaterally. On average, there was an increase in the DBRN surface facing the vessel (surface of contact) at the level of the muscular branch compared to the level of the RRAab, for both supination and pronation (Fig. 7B). There was a decrease in the diameter of the muscular branches compared to the diameter of the RRAab (Fig. 7C). However, no significant change was observed in the vessel diameter by changing the position of the forearm from supination to pronation. On the left side, there was a decrease in the mean distance between the muscular branch and the DBRN compared to the distance between the RRAab and the DBRN in supination and pronation (Fig. 7D). On the right side, the decrease in the nerve-vessel distance at the level of the muscular branch compared to the level of the RRAab did not reach significance. There was a tendency for better visualization of the fat plane between the vessels of the LoH and DBRN by changing the forearm position from supination to pronation.

There was a positive correlation between the diameter of the vessels of the LoH and weight, height, and BMI in both supination and pronation bilaterally (RRAab right side supination/pronation: *p* = 0.001/0.002, *p* < 0.001/0.001, *p* = 0.043/0.035, RRAab left side supination/pronation: *p* ≤ 0.01/0.01, *p* = 0. 001/0.007, *p* ≤ 0.016/0.001, muscular branch right side supination/pronation: *p* = 0.006/0.005, *p* = 0.027/0.009, *p* = 0.006/0.013, muscular branch left side supination/pronation: *p* ≤ 0.006/0.001, *p* = 0.003/0.020, *p* = 0.048/0.002, respectively). In addition, there was a positive correlation between the percentage of DBRN surface facing the LoH and height on the left side (RRAab supination/pronation: *p* ≤ 0.001/0.001, muscular branch supination/pronation: *p* = 0.001/0.024). Additional correlations observed in only one of the vessels of the LoH, one position of the forearm, or one side of the body are displayed in the supplemental material.

## Discussion

This study conducted a detailed assessment of the neurovascular crossing patterns between the LoH and the DBRN in two different positions of the forearm, using HRUS, color Doppler, and MRI with anatomic correlation. We confirmed our initial hypothesis, revealing a predominant pattern where the RRAab crosses below and the muscular branch crosses above the DBRN, accounting for approximately 85% and more than 63% of cases, respectively. From an interventional standpoint, our study highlights the high incidence of the muscular branch crossing above the DBRN, serving as a critical alert for interventional radiologists accessing the radial tunnel. In addition, our study was the first to meticulously map the DBRN surfaces most prone to vascular compression, shedding light on a mechanism of partial nerve lesions.

The neurovascular crossing patterns were evaluated in both ex vivo and in vivo studies. An advantage of the ex vivo study was the ability to directly compare specimen images with dissection findings, as well as the mitigation of motion artifacts that can prolong exams due to the need for repeated scans. Conversely, in the in vivo study, muscle tone was maintained, and vessel distension and flow were preserved, providing a more accurate representation of the dynamic relationships between the structures. However, MRI scans were not performed in the in vivo study, partly due to higher costs and participants’ time constraints.

The RRAab has been noteworthy for its participation in the arterial network supply of the elbow, anastomosing with the descending radial collateral artery [11]. The other vessels of the LoH have been described as traversing several intermuscular layers, typically following a transverse path to provide vascular supply to the muscles of the lateral compartment of the forearm [1]. The DBRN and its continuation into the supinator tunnel as the PIN can be compressed at any anatomic site along its path. However, the LoH is one of the five most relevant potential sites for nerve entrapment, along with fibrous bands in front of the radial-capitellar joint, the tendinous margin of the extensor carpi radialis brevis, the arcade of Frohse (thickened tendinous arch at the superior margin of the superficial head of the supinator muscle), and the inferior margin of the supinator muscle [12–15]. Despite the relevance of LoH, there is a lack of detailed knowledge of its relationship with DBRN, which is important for understanding entrapment syndromes and guiding interventional procedures.

Interventional procedures, such as dry needling and hydrodissection, have emerged as alternatives to open surgery for treating entrapment syndromes of the DBRN and PIN [16–21]. These procedures are often performed blindly or under HRUS guidance and demand a robust knowledge of the anatomy of the radial tunnel to avoid complications. Dry needling has gained popularity among physical therapists as a treatment for myofascial pain syndrome [19, 22]. To the best of our knowledge, at the time of this article’s publication, only one cadaveric study and one case report in the English language explored the use of dry needling for radial tunnel syndrome. Both studies focused on needle insertion at the supinator muscle belly, which is outside the scope of our current study.

In contrast, hydrodissection aims to create non-existent surgical planes by introducing a solution around the nerve that temporarily removes adhesions and releases nerve pressure [17, 23]. This solution can be saline, anesthetics, corticosteroids, dextrose, or platelet-rich plasma. Compared to dry needling, hydrodissection has been described in a greater number of patients for the treatment of radial tunnel syndrome. In a recent review, Gill et al. highlighted the use of hydrodissection in 75 patients with radial tunnel syndrome, with the patients pulled from case series and case reports [20].

A best practice in performing hydrodissections of peripheral nerves is to carefully identify vessels in the needle’s path to prevent unintended vessel injection and vessel perforation with hematoma formation. For the DBRN, our study demonstrated that most often, the muscular branch is the vessel that can be interposed between the needle and the nerve via the anterior approach in the positions of supination and pronation. The needle is typically placed at the entrapment area, usually in the region of the arcade of Frohse, the most common site of entrapment. The arcade of Frohse is close to the muscular branch of the LoH, thus increasing the risk of vascular injury. Interestingly, we observed that altering the forearm position from supination to pronation tended to improve the visualization of the fat plane separating the nerve from the vessel. This simple change in forearm positioning can be a valuable strategy for optimizing the visualization of these critical anatomical structures.

In our series, we consistently observed a crossing between the RRAab and the DBRN in all cases. The incidence of crossover and cross-under patterns for this vessel was similar in both in vivo and ex vivo, with cross-under, the predominant pattern, occurring in 83.3% of ex vivo cases and 85.3% of in vivo cases. In contrast, the crossing patterns of the muscular branch exhibited greater variability in vivo. While the crossover pattern was prevalent in 100% of ex vivo cases, it was observed in 63.2% of in vivo cases. Additionally, we encountered instances where no crossing between the muscular branch and the DBRN was identified, which accounted for 8.8% of in vivo cases. We speculate that these findings may be attributed to undetected small-caliber vessels and/or anatomical variations, which are likely more common at the muscular level than the RRAab level. Moreover, such nuances might be better discerned in vivo due to the larger sample size.

Our study suggests that both the deep and superficial surfaces of the DBRN may be vulnerable to mechanical irritation from the LoH. Notably, statistical analysis indicated that the deep surface is more prone to mechanical irritation by the RRAab, while the superficial surface is more prone to irritation by the muscular branch. However, in a proportion of cases (38.3%), both vessels were observed to pose a risk to the same nerve surface. This occurrence hypothetically doubles the risk to that surface, potentially amplifying the likelihood of fascicular damage. Specifically, the pattern of both vessels crossing above the nerve accounted for 11.8%, while both vessels crossing below accounted for 26.5%. In summary, our findings suggest an increased susceptibility to mechanical irritation of the superficial surface of the DBRN at the level of the muscular branch. This susceptibility is attributed to the vessel crossing pattern, changes in nerve morphology, and increased surface contact between the nerve and vessel at this level. Although infrequent, this susceptibility may be exacerbated by the occurrence of the RRAab crossing above the nerve.

As expected, our investigation revealed a positive correlation between vessel diameter and anthropometric factors such as weight, height, and BMI in our in vivo cases, which was consistent bilaterally and across the positions of supination and pronation of the forearm. This finding indicates a proportional relationship between vessel size and body structure for the LoH. However, unlike conventional arteries, the RRAab stands out by originating distally and following an ascending path. Then, upon comparing the diameter of vessels in the LoH, we noted that the muscular branch exhibited a significantly smaller diameter than the RRAab, which branches off later. Interestingly, in our series, the RRAab appeared more as a continuation of the trunk of the radial recurrent artery than as a smaller branch.

Despite the smaller diameter of the muscular branch, our analysis revealed that the proportion of the DBRN surface facing the vessels of the LoH increased at the level of the muscular branch compared to the RRAab for both supination and pronation. During image analysis, the crossings between the RRAab and DBRN mainly occurred at the lateral edge of the brachialis muscle, where the local fat pad was visibly wider. In contrast, the crossings between the muscular branch and DBRN occurred predominantly at the proximal portion of the deep head of the supinator muscle (pre-arcade of Frohse), where the fat pad was relatively smaller. We speculate that this increase in contact surface is attributed to a greater flattening of the nerve at the level of the muscular branch, possibly due to increased pressure exerted by adjacent muscular structures, thereby expanding the potential nerve-vessel contact.

This study has some limitations. First, the surfaces of the nerve are not fixed, and some variation is expected with changes in the position of the forearm. Despite advances in characterizing the somatotopic fascicular organization of various peripheral nerves, there is still limited information on the internal topography of the DBRN/PIN [24, 25]. Sunderland’s remarkable work in 1945 described in detail the radial nerve fascicular arrangement from distal to proximal, starting from the point of division at the elbow to approximately the level of the brachio-axillary angle. However, according to the author, the reduction of the posterior interosseous bundles into a single funiculus hindered the examination of the topography of the posterior interosseous branches within the radial trunk. Subsequent studies of DBRN and PIN have primarily focused on elucidating the anatomic branching patterns and conducting histological analysis to quantify myelinated fibers for nerve transfer procedures, rather than delving into the intricate topography of the nerve’s fascicles [24, 26]. Therefore, future investigations should focus on an in-depth analysis of the fascicular arrangement within the DBRN/PIN, including an analysis in different positions of the forearm, to establish a more accurate correlation with the potential areas of vulnerability described in our study. Second, the sample size of both in vivo and ex vivo cases was relatively modest, and it is plausible that other anatomical variations not discerned in our study may exist. Finally, while our study delves into the relationship between the LoH and the DBRN, it is important to note that the SBRN also interacts with other muscular vessels of the LoH, a facet not explored in our research. Future studies could investigate this aspect to better understand other neurovascular interactions in the radial tunnel region.

In conclusion, the crossing pattern of the RRAab below and the muscular branch above the DBRN accounted for the vast majority of cases. Both the deep and superficial surfaces of the DBRN are vulnerable to mechanical irritation by the vessels of the LoH. However, anatomic features potentially increase the chances of mechanical irritation at the level of the muscular branch and, therefore, the superficial surface of the nerve. Knowledge of neurovascular crossings is crucial for understanding neurovascular entrapment syndromes and planning interventional procedures to reduce complications of vascular origin.

## Supplementary Information

Below is the link to the electronic supplementary material.

Supplementary file1 (DOCX 1792 KB)

## Data Availability

The authors declare that the data supporting the findings of this study are available within the paper and its supplementary information files. Additional data sets generated during the current study are available from the corresponding author upon reasonable request.
