# Supplementary material for: Neurovascular crossing patterns between leash of Henry and deep branch of radial nerve: implications for neurointervention and diagnostic imaging
Source: Skeletal Radiol. 2024 Jul 31;54(3):493–507. doi: 10.1007/s00256-024-04740-1 (PMC11769873; doi:10.1007/s00256-024-04740-1)
Supplement: Supplementary file 1 — Supplementary file1 (DOCX 1792 KB) [file 256_2024_4740_MOESM1_ESM.docx]

**Supplemental material.**


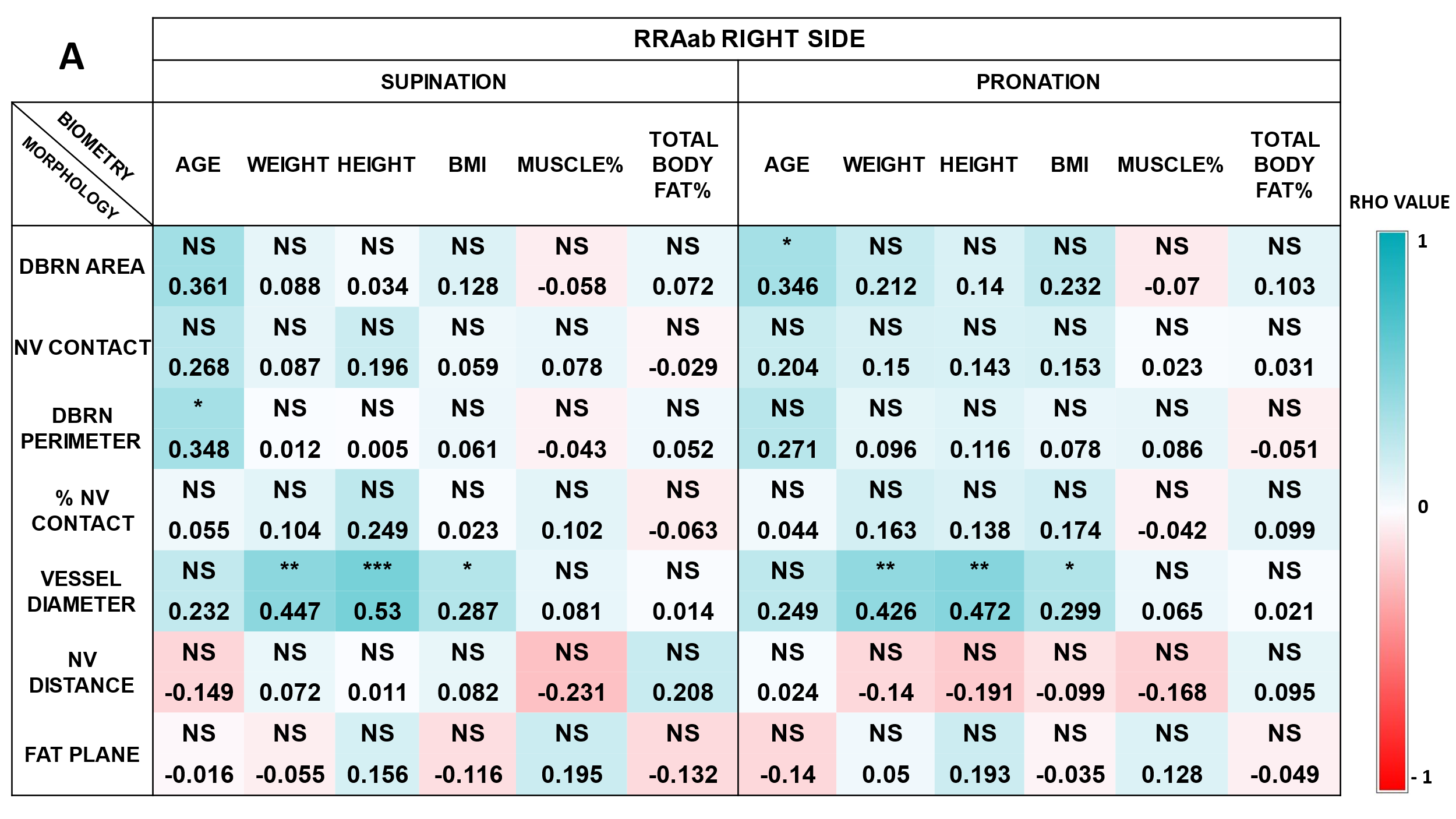

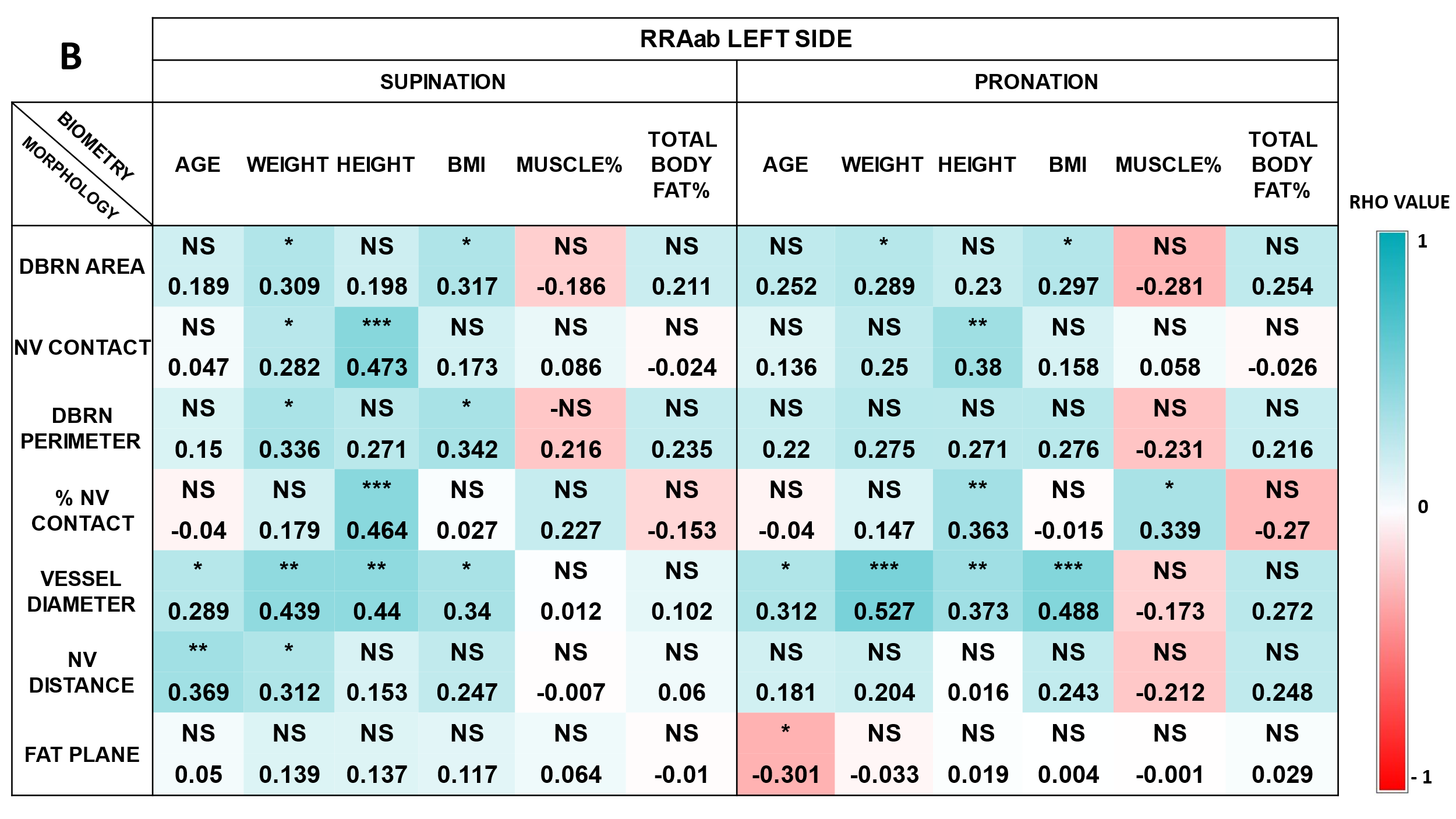

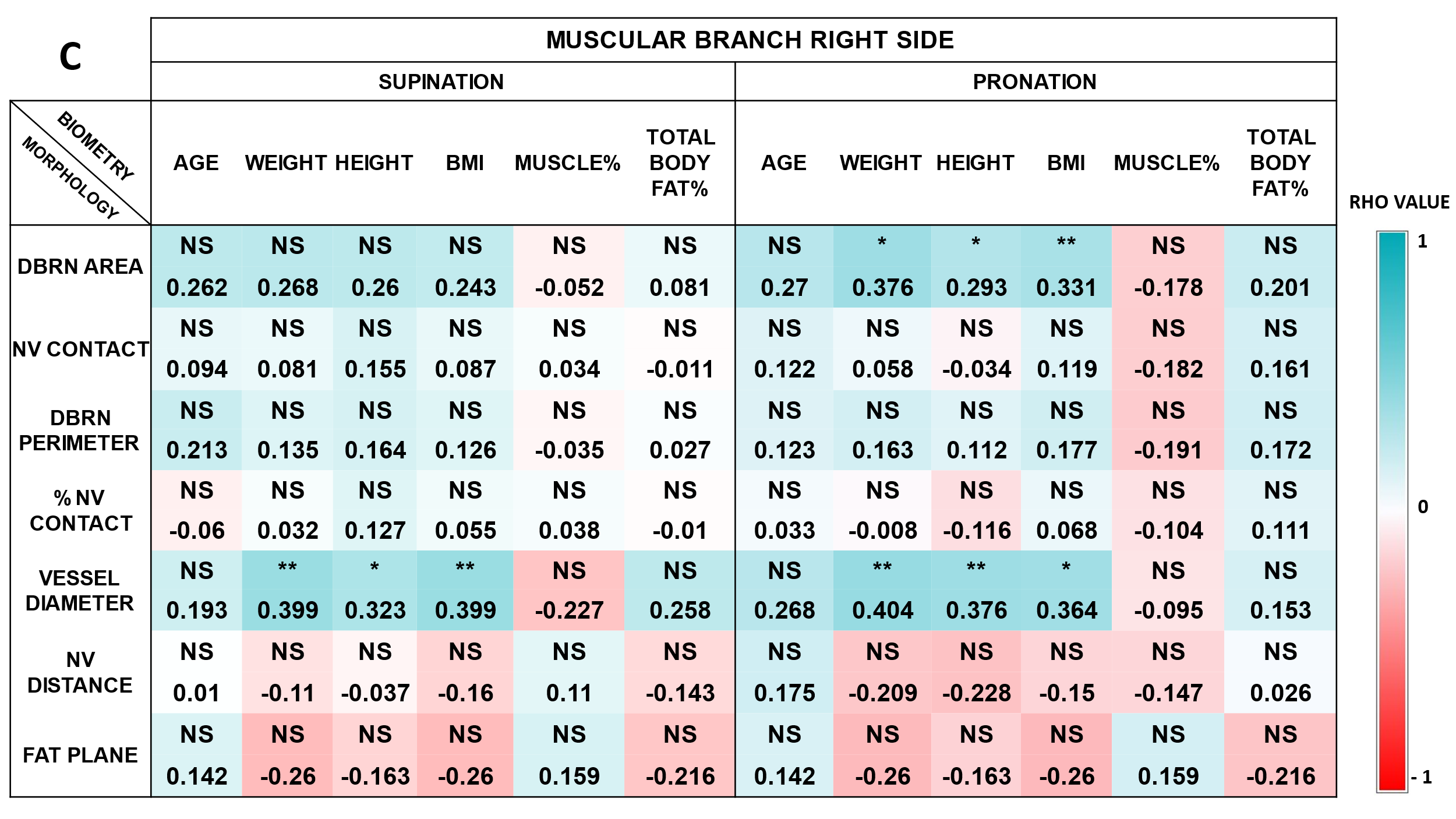

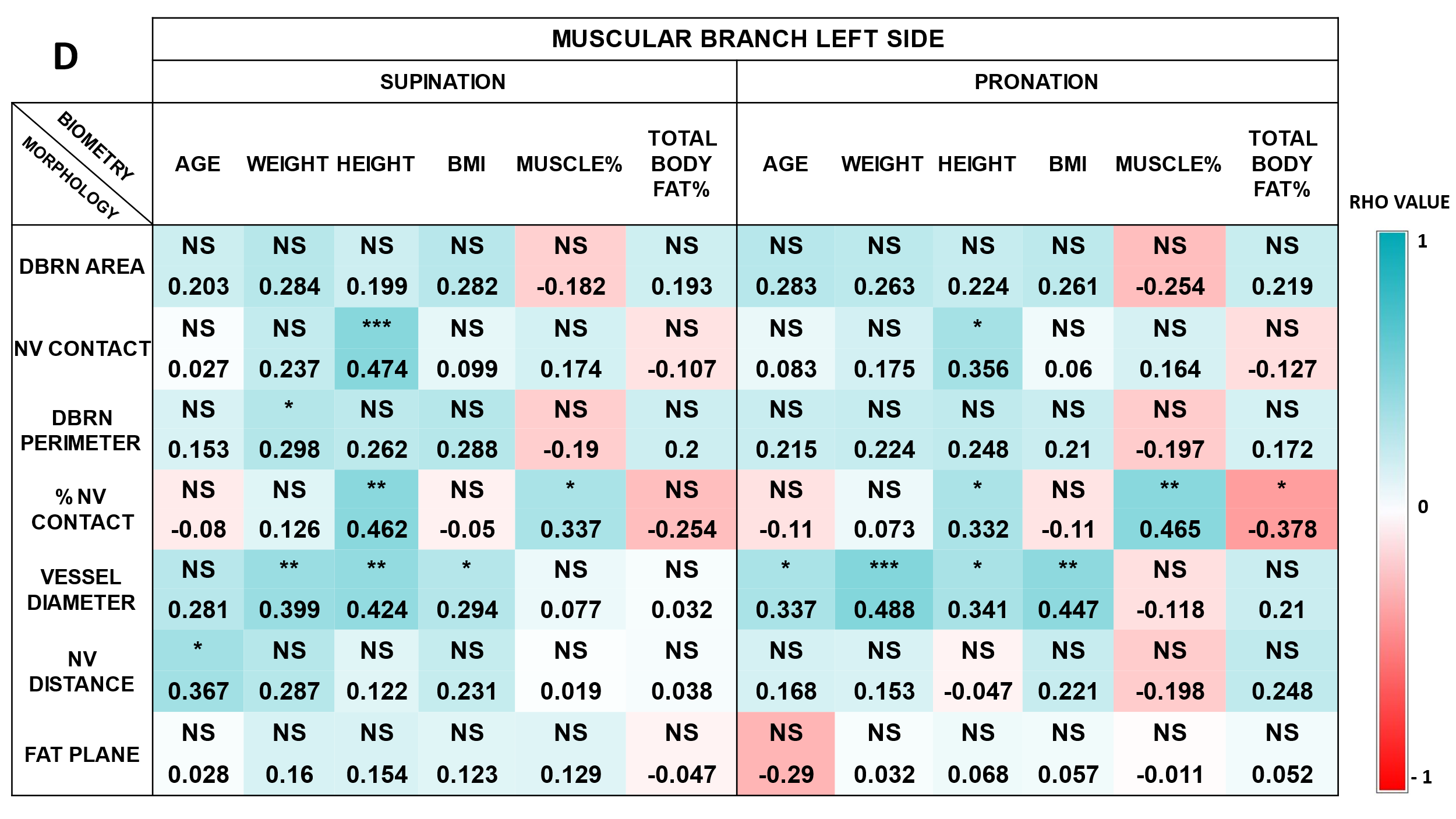


Correlation between biometric features and morphology of the deep branch of the radial nerve and vessels of the leash of Henry. **A,B** Level of the ascending branch of the radial recurrent artery (RRAab). **C,D** Level of the muscular branch. First line: p value. Second line: Rho value. * p < .05, ** p < .01, *** p < .001.
